# Mini-laparoscopy as a diagnostic tool for abdominal tuberculosis: a retrospective series of 29 cases

**DOI:** 10.1007/s00464-022-09703-y

**Published:** 2022-10-13

**Authors:** Thomas Theo Brehm, Natascha Ndzedzeka-Völz, Malte Wehmeyer, Martin Christner, Till Sebastian Clauditz, Peter Hübener, Marylyn M. Addo, Ansgar W. Lohse, Stefan Schmiedel

**Affiliations:** 1 I. Department of Internal Medicine, University Medical Center Hamburg-Eppendorf, Martinistraße 52, 20246 Hamburg, Germany; 2 German Center for Infection Research (DZIF), Partner Site Hamburg-Lübeck-Borstel-Riems, Hamburg, Germany; 3 Institute of Medical Microbiology, Virology and Hygiene, University Medical Center Hamburg-Eppendorf, Martinistraße 52, 20246 Hamburg, Germany; 4 Department of Pathology, University Medical Center Hamburg-Eppendorf, Martinistraße 52, 20246 Hamburg, Germany; 5 Institute for Infection Research and Vaccine Development (IIRVD), University Medical Center Hamburg-Eppendorf, Martinistraße 52, 20246 Hamburg, Germany

**Keywords:** Diagnosis, Laparoscopy, Tuberculosis

## Abstract

**Objectives:**

Abdominal tuberculosis (TB) is a “great mimic,” and diagnosis remains challenging even for experienced clinicians. While mini-laparoscopy has already been demonstrated to be an efficient diagnostic tool for a variety of diseases, we aimed to demonstrate the feasibility of this technique in diagnosing abdominal TB.

**Methods:**

We retrospectively included patients who underwent mini-laparoscopy at the University Medical Center Hamburg-Eppendorf between April 2010 and January 2022 for suspected abdominal TB. Demographic, clinical, and laboratory data, radiological findings as well as macroscopic, histopathologic, and microbiologic results were analyzed by chart review.

**Results:**

Out of 49 consecutive patients who underwent mini-laparoscopy for suspected abdominal TB, the diagnosis was subsequently confirmed in 29 patients (59%). Among those, the median age was 30 years (range 18–86 years) and the majority were male (*n* = 22, 76%). Microbiological diagnosis was established in a total of 16 patients. The remaining patients were diagnosed with abdominal TB either by histopathological detection of caseating granulomas (*n* = 3), or clinically by a combination of typical presentation, mini-laparoscopic findings, and good response to anti-tuberculous treatment (*n* = 10). Bleeding from the respective puncture site occurred in 19 patients (66%) and either resolved spontaneously or was arrested with argon plasma coagulation alone (*n *= 10) or in combination with fibrin glue (*n *= 1). Minor intestinal perforation occurred in 2 patients and was treated conservatively.

**Conclusions:**

Mini-laparoscopy is a useful and safe modality for the diagnosis of abdominal TB.

**Supplementary Information:**

The online version contains supplementary material available at 10.1007/s00464-022-09703-y.

Tuberculosis (TB) is a communicable disease caused by the bacteria *Mycobacterium tuberculosis*. It generally affects the lungs but can also involve almost any other organ. Those extrapulmonary manifestations accounted for around 16% of the 7.1 million incident cases of TB that were notified in the year 2019 worldwide [1]. One of the most common forms of extrapulmonary TB is abdominal TB, which may affect the gastrointestinal tract, lymph nodes, peritoneum, and visceral organs [2, 3]. Abdominal TB has likely become more prevalent as a result of the HIV/AIDS epidemic since HIV-infected patients are more likely to present with extrapulmonary TB manifestations [4]. Clinically overt abdominal TB can result from ingestion of infected sputum or milk, as a reactivation of latent TB infection, or by hematogenous or lymphatic dissemination in the setting of active pulmonary TB or miliary TB [2]. The clinical presentation of abdominal TB is generally non-specific and may mimic various conditions like inflammatory bowel disease [5], abdominal malignancies [6], or other infectious diseases [7]. Since laboratory features are also not characteristic, the diagnostic process is often challenging and requires a high index of clinical suspicion. Abdominal ultrasound and CT are very useful tools in the diagnostic process, but oftentimes fail to reach the final diagnosis with sufficient certainty [8, 9]. Conventional diagnostic laparoscopy allows for macroscopic evaluation of the abdominal cavity and offers the opportunity of targeted biopsies of focal lesions for histopathologic and microbiologic analyses under visual control. As an alternative to conventional laparoscopy, mini-laparoscopy uses small-diameter trocars and instruments and can be performed under conscious sedation [10]. While this technique has been demonstrated to be a relatively safe and effective method for the evaluation of different hepatic, splenic, and peritoneal pathologies [11–17], there have been only few single case reports on the diagnostic value in patients with abdominal TB [11, 18]. In the present study, we aimed to investigate the feasibility and safety of mini-laparoscopy in the diagnostic process of peritoneal and visceral forms of abdominal TB.

## Materials and methods

### Study population and data collection

All patients who underwent mini-laparoscopy for suspected abdominal TB at the University Medical Center Hamburg-Eppendorf between April 2010 and January 2022 were included in the analysis. Abdominal TB was either diagnosed by positive microscopy, mycobacterial culture, or *M. tuberculosis* PCR (microbiological diagnosis), by detection of caseating granulomas in tissue biopsies (histopathological diagnosis), or by a combination of a typical presentation, mini-laparoscopic findings, and good response to anti-tuberculous treatment (clinical diagnosis). We assessed age, gender, comorbidities, symptoms, suspected diagnosis on admission, laboratory parameters, radiological, microbiological, and histopathological results, macroscopic findings, complications, and disease course from electronic patient records by retrospective chart review. The study was reviewed and approved by the Ethics Committee of the Medical Council of Hamburg (WF-185/20). Data were analyzed utilizing descriptive statistics.

### Mini-laparoscopy

Mini-laparoscopy and guided biopsies were performed as previously described [16] using a 2.75 mm trocar, a 2.3 mm Veress needle, and a 1.9 mm optic laparoscope (Fa. Wolf GmbH, Knittlingen, Germany). Nitrous oxide (N_2_O) was used as insufflation gas. All procedures were performed under conscious sedation with propofol, midazolam, and pethidine, as well as local anesthesia with lidocaine.

### Microbiological methods

Interferon-γ release assay (IGRA) was performed using the QuantiFERON-TB Gold assay (Cellestis, GmbH, Statens Serum Institute, Copenhagen, Denmark) as recommended by the manufacturer. Results were expressed in international units (IU) of the interferon-γ concentration with ≥ 0.35 IU/ml defined as positive. Smears were investigated for acid-fast bacilli with Ziehl–Neelsen staining and light microscopy and graded according to standard criteria. Mycobacterial culture was performed from NALC–NaOH-decontaminated samples using liquid (BACTEC MGIT 960 mycobacteria growth indicator tube; Becton Dickinson, Franklin Lakes, NJ, USA) and solid (Löwenstein-Jensen, Stonebrink) media, incubated for up to 6 and 8 weeks, respectively, with subsequent identification by PCR or rpoB-sequencing. Mycobacterium tuberculosis DNA was detected with the Xpert MTB/RIF Ultra test (Cepheid, CA, USA) according to the manufacturer's instructions.

## Results

### Baseline characteristics of the study population

**Fig. 1:**
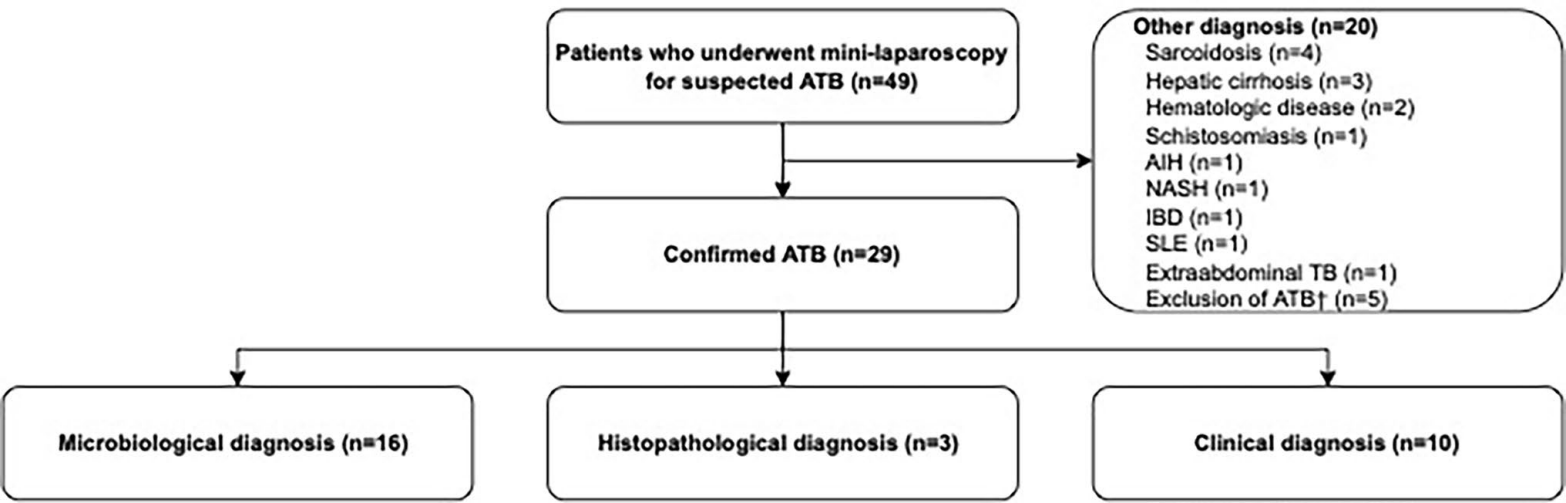
Flowchart of the study population. *AIH* autoimmune hepatitis; *ATB* abdominal tuberculosis; *IBD* inflammatory bowel disease; *NASH* non-alcoholic steatohepatitis; *SLE* systemic lupus erythematosus; *TB* tuberculosis; ^†^In 3 patients, abdominal tuberculosis was excluded but no definite diagnosis was established

During the study period, 49 consecutive patients underwent mini-laparoscopy for suspected abdominal TB. Of those, the diagnosis was confirmed in 29 patients (59%), and the rest was diagnosed with various other conditions (Fig. 1). Baseline characteristics of patients with confirmed abdominal TB are presented in Table 1. The median age was 30 years (range: 18–86 years), and the majority of patients were male (*n* = 22, 76%). Most patients were migrants from sub-Saharan Africa (*n* = 17, 59%), South and Southeast Asia (*n* = 5, 17%), Western Asia (*n* = 3, 10%), South America (*n* = 1, 3%), or the Caribbean (*n* = 1, 3%). Two patients (7%) had no history of migration. Six patients (21%) had an HIV infection and 3 patients (10%) suffered from chronic liver disease. A total of 5 patients (17%) had been diagnosed with pulmonary TB before the diagnosis of abdominal TB was established. The most common symptoms on admission were fever (*n* = 20, 69%), followed by weight loss (*n* = 16, 55%), abdominal pain (*n* = 16, 55%), and night sweats (*n* = 14, 48%). Fewer patients reported malaise (*n* = 10, 34%), cough (*n* = 8, 28%), inappetence (*n* = 7, 24%), chills (*n *= 6, 21%), diarrhea (*n* = 5, 17%) or nausea, and vomiting (*n* = 3, 10%).

**Table 1:** Baseline characterization of the study population

Gender, *n* (%)	
Male	22 (76)
Female	7 (24)
Age	
Median	30
Range	18–86
Origin, *n* (%)	
Subsaharan Africa	17 (59)
South and Southeast Asia	5 (17)
West Asia	3 (10)
Europe	2 (7)
South America	1 (3)
The Carribean	1 (3)
Medical history, *n* (%)	
HIV	6 (21)
Pulmonary tuberculosis	5 (17)
Chronic liver disease	3 (10)
Symptoms, *n* (%)	
Fever	20 (69)
Weight loss	16 (55)
Abdominal pain	16 (55)
Night sweats	14 (48)
Malaise	10 (34)
Cough	8 (28)
Inappetence	7 (24)
Chills	6 (21)
Diarrhea	5 (17)
Nausea and vomiting	3 (10)

*HIV* Human immunodeficiency virus

### Laboratory results of patients with abdominal TB

Laboratory parameters of all patients with confirmed abdominal TB on hospital admission are presented in Supplemental Table 1. Most patients (*n *= 24, 83%) presented with moderate anemia. All patients had elevated C-reactive protein (CRP) levels (median: 92 mg/L, IQR: 67–115 mg/L), and, when tested, erythrocyte sedimentation rate (ESR) (median: 59 mm/h, IQR: 36–77 mm/h). Increased levels of alanine aminotransferase (ALAT) were observed in 41% of patients (*n* = 12, median: 31 U/L, IQR: 18–56 U/L).

### Radiological findings and macroscopic findings of mini-laparoscopy of patients with abdominal TB

**Fig. 2:**
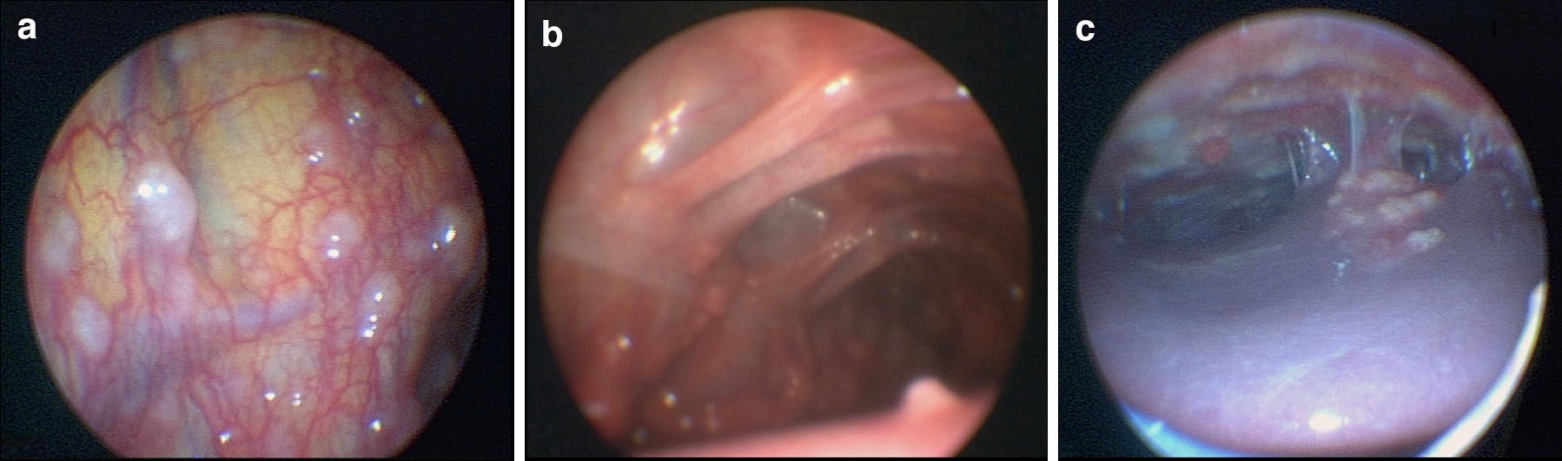
Mini-laparoscopic view of abdominal tuberculosis. Mini-laparoscopic view of abdominal tuberculosis showing multiple whitish granular nodules scattered over the peritoneum (**a**, **b**, **c**) and the liver **c** and thickened intraabdominal adhesions **b**, **c**

Abdominal ultrasound and abdominal computed tomography (CT) scans were performed on 26 (90%) of all patients with abdominal TB. The respective results as well as the macroscopic findings detected by visual inspection of the peritoneum and visceral organs during mini-laparoscopy are presented in Table 2 and Fig. 2. Mini-laparoscopic visualization of the liver and the peritoneum was possible in all patients, but visualization of the spleen was not possible in 14 (48%) patients due to intraabdominal adhesions. While mini-laparoscopy identified suspicious focal hepatic lesions in 15 (52%) patients, only six of those lesions were detected by abdominal CT and only one lesion by abdominal ultrasound. Focal splenic lesions were described in seven patients by abdominal CT and abdominal ultrasound, respectively. Focal splenic lesions were detected in six (21%) patients. Suspicious peritoneal lesions were observed during mini-laparoscopy in 17 (59%) patients. Of those patients, 15 patients underwent abdominal CT, which only detected five of those lesions. Abdominal ultrasound did not detect any focal peritoneal lesions. Ascites was detected in 14 (54%) patients by abdominal ultrasound and in 12 (46%) patients by abdominal CT. In addition, abdominal lymphadenopathy was detected by abdominal ultrasound and CT in 10 (38%) and 12 (46%) patients, respectively.

**Table 2:** Microbiological and histopathological results from tissue samples obtained by mini-laparoscopy

	Total	Liver	Peritoneum	Spleen
Microbiological results				
Total (examined)—*n*	28	20	13	4
Total (positive)—*n* (%)	16 (57)	7 (35)	10 (77)	0 (0)
Microscopy—*n* (%)	1 (6)	1 (14)	0 (0)	0 (0)
Mycobacterial culture—*n* (%)	15 (94)	6 (86)	10 (100)	0 (0)
PCR—*n* (%)	13 (81)	5 (71)	9 (90)	0 (0)
Histopathological results				
Total (examined)—*n*	26	21	13	5
Caseating granuloma—*n* (%)	14 (54)	5 (24)	9 (69)	2 (40)
Non-caseating granuloma—*n* (%)	10 (38)	9 (43)	2 (15)	1 (20)

*PCR* polymerase chain reaction

### Microbiological results of patients with abdominal TB

An IGRA was performed on 21 patients with abdominal TB, of whom 13 (62%) had a positive test result. During mini-laparoscopy, at least one specimen for microbiological analyses was obtained from 28 (97%) patients (Table 3 and Supplemental Table 2). In total, 20 samples from the liver, 13 samples from the peritoneum, 4 samples from the spleen, and one ascitic sample were analyzed. In 16 patients abdominal TB was subsequently microbiologically confirmed by positive microscopy (*n* = 1), mycobacterial culture (*n *= 15), and/or PCR for *M. tuberculosis* DNA (*n* = 13). Those positive PCR results were obtained from fresh native tissue specimens (*n *= 11/23, 48%) and/or from formalin-fixed, paraffin-embedded tissue specimens (*n* = 8/19, 42%).

**Table 3:** Radiological results and macroscopic findings observed during mini-laparoscopy

	Mini-laparoscopy	Ultrasound	CT scan
Total—*n*	29	26	26
Any lesions—*n* (%)	28 (97)	8 (31)	13 (50)
Peritoneal lesions—*n* (%)	17 (59)	–	5 (19)
Hepatic lesions—*n* (%)	15 (52)	1 (4)	6 (23)
Splenic lesions—*n* (%)	6 (21)	8 (31)	7 (27)

*CT* computed tomography

### Histopathological findings of patients with abdominal TB

Tissue specimens for histopathologic evaluation were obtained during mini-laparoscopy from 26 (90%) patients with abdominal TB (Table 2). Caseous necrosis was detected in 14 and non-caseating granulomas in 10 patients. In total, 3 patients with negative microbiological results were diagnosed with TB by detection of caseous necrosis.

### Safety of mini-laparoscopy procedures

Out of 29 liver biopsies that were performed, post-puncture bleeding was observed in 24 (82%) cases, which either resolved spontaneously (*n* = 13) or was arrested by APC (argon plasma coagulation) (*n* = 11) (Supplemental Table 3). Five spleen biopsies were performed, and the respective post-puncture bleedings were arrested either by APC alone (*n* = 4) or by a combination of APC and fibrin glue (*n* = 1). Out of 17 peritoneal biopsies, minor post-biopsy bleeding was only observed in one case, which resolved spontaneously. Procedural intestinal perforation occurred in 2 patients. Both of them were closely monitored clinically and received intravenous antibiotics but did not develop any signs and symptoms related to the perforation and did not require surgical interventions. Post-puncture bleeding was noticed in 15 out of the 17 patients with initially suspected but not confirmed abdominal TB, which stopped spontaneously in 9 patients and after APC in 6 patients. There was no need for secondary interventions or surgery, admission to the intensive care unit, and no deaths among patients with confirmed or suspected TB.

## Discussion

This study is the largest case series of patients with abdominal TB diagnosed by mini-laparoscopy. Four patients with splenic tuberculosis in this case series are also part of a previously published study by our group on the effectiveness and safety of mini-laparoscopy-guided spleen biopsy [11]. To the best of our knowledge, there are no other case series on the feasibility of mini-laparoscopy in the diagnosis of abdominal TB to date. Our results confirm earlier reports about the difficulties of diagnosing abdominal TB due to the protean and insidious nature of clinical symptoms and the lack of specific laboratory, radiological, or clinical findings [3].

In line with previous studies [19], the most consistent laboratory features in patients with abdominal TB were anemia, elevated CRP, and elevated ESR. These features are highly unspecific and not able to distinguish abdominal TB from the wide range of differential diagnoses. Since neither tuberculin skin tests nor IGRAs can accurately discriminate between latent and active disease, they are not recommended for the diagnosis of active TB in low- and middle-income countries [20]. Also, IGRAs cannot provide sufficient sensitivity for the diagnosis of extrapulmonary TB [21], which is underlined by the finding that less than half of patients in our study had a positive test result. While radiological investigations may play an important role in the diagnosis of both pulmonary and extrapulmonary forms of TB, our study also highlights the limitations of CT and ultrasound and demonstrates the overall diagnostic value of mini-laparoscopy in patients with abdominal TB. CT is generally regarded as the most informative radiological modality in the diagnosis of abdominal manifestations and CT abnormalities have been reported in 69% to 78% of patients with visceral or peritoneal TB [8, 22]. Common findings are often nonspecific and include thickening of the peritoneum, ascites, lymphadenopathy, hepatomegaly, hepatic, or splenic lesions, and calcifications [8, 22, 23]. It has been proposed that PET/CT imaging may be useful in diagnosing extrapulmonary TB [24], but this warrants further investigation in larger studies. Besides, the cost and accessibility of this imaging modality limit its utility in resource-limited TB-endemic settings. Abdominal ultrasound is usually the first imaging study obtained in the assessment of patients with abdominal symptoms. A meta-analysis including 879 HIV-positive individuals with abdominal TB recently showed that abdominal ultrasound has a diagnostic sensitivity of 63% for any abnormality [9]. It has therefore been proposed to be a feasible diagnostic tool in the investigation of patients with suspected abdominal TB in resource-limited healthcare settings with a high pre-test probability for TB [9, 25]. Remarkably, in our study, neither abdominal ultrasound nor abdominal CT detected the majority of focal lesions that were observed by visual inspection of the peritoneum and visceral organs during mini-laparoscopy.

While clinical, laboratory, and radiological findings can raise suspicion of abdominal TB, tissue biopsies are generally required to make a definite diagnosis and fresh native tissue specimens should be sent for AFB stain, mycobacterial culture and *M. tuberculosis* PCR. Mycobacterial culture is essential since it is the only diagnostic modality to reliably allow for drug susceptibility testing to exclude drug-resistant TB. In our cohort, mycobacterial culture of tissue samples obtained during mini-laparoscopy grew *M. tuberculosis* in 15 patients, one of which was resistant to isoniazid. While mycobacterial culture is paramount to perform susceptibility testing and thus ensure effective treatment, it has a low diagnostic yield of only 10 to 35% for abdominal TB, and the results may require several weeks due to the slow growth rate of *M. tuberculosis* [26]. AFB stain has an even lower sensitivity ranging between 3 and 25% [4, 26]. Tissue PCR from fresh native tissue specimens has been demonstrated to be the most specific diagnostic test for abdominal tuberculosis with an overall sensitivity of 58% to 86% [4, 27]. The classic histology of caseating granulomas can be found in 51 to 83% of patients with hepatic TB [28], 76 to 100% of patients with tuberculous peritonitis [29–32], and the majority of reported cases of splenic TB [33, 34]. Our study highlights that no single diagnostic method reliably identifies all cases of abdominal TB and can serve as a sufficiently sensitive gold standard. Rather, a combination of microbiological results, histopathological findings, and a good response to anti-tuberculous treatment should be used to diagnose abdominal TB. The frequent discrepancies between microbiological and histological results from tissue samples may be explained by the fact that not all examinations were necessarily performed on the same specimen and by a heterogeneous distribution of mycobacteria in infected organs. Therefore, ideally several tissue biopsies should be obtained for both microbiological and histopathological analyses. In patients with suspected abdominal TB, conventional laparoscopy has been considered the investigation of choice. It presents the opportunity to visualize the surface of the liver, the spleen, and the peritoneal cavity and offers the option of obtaining targeted tissue specimens from suspicious focal lesions [35]. Mini-laparoscopy represents a cheaper and less invasive alternative to conventional laparoscopy which requires smaller insertions to the abdominal wall due to ultra-fine instrumentation and can be safely conducted outside of the operation room with the patient under conscious sedation. Compared to percutaneous fine-needle aspiration biopsies, laparoscopy allows targeted core needle biopsies with the acquisition of larger tissue samples, which may be required to perform not only reliable histological analyses but also microbiological culture and *M. tuberculosis* PCR. Several studies demonstrated a high diagnostic yield of more than 80% in patients with suspected abdominal TB [26, 35]. In addition, while percutaneous biopsies are considered by many to be contraindicated in patients with high bleeding risk, laparoscopic techniques can be performed safely even in patients with severe coagulopathies [16] and enable termination of post-biopsy bleeding with various coagulation methods. This is of particular relevance for patients with suspected abdominal TB since mycobacterial infection has been identified as a significant risk factor for bleeding complications after percutaneous liver biopsy [36]. In our study cohort, minor post-biopsy bleeding occurred in 19 (66%) patients and was subsequently successfully controlled using argon plasma coagulation, fibrin glue, or both. Since abdominal TB is virtually always associated with intra-abdominal adhesions, laparoscopic techniques have a certain risk of intestinal perforation. In our cohort, intestinal perforation occurred in 2 patients, neither of whom required secondary interventions or admission to the ICU. These uncomplicated clinical courses demonstrate that mini-laparoscopy is a safe alternative to conventional laparoscopy due to the ultra-fine instrumentation. However, these findings also highlight that mini-laparoscopy should only be conducted by experienced physicians at hospitals equipped to handle complications that may potentially arise. Intraabdominal adhesions may hypothetically limit the feasibility of laparoscopic techniques since visualization may be impaired and biopsies may not be carried out. In our cohort visualization of the liver and the peritoneum was possible and tissue biopsies could be performed in all individuals. However, visualization of the spleen was only possible in around half the patients, so isolated splenic lesions may potentially be missed. While mini-laparoscopy has already been demonstrated to be relatively safe and efficient in the diagnosis and staging of a variety of hepatic [10, 12, 14–16], splenic [11, 17], and peritoneal [13] diseases, our study provides evidence that this technique can also be useful in the diagnostic work-up of patients with suspected abdominal TB.

Our study is subject to several limitations that are mainly inherent to the retrospective study design. Firstly, we included patients that received mini-laparoscopy for suspected abdominal TB but no control group that was diagnosed by other means. While we demonstrate the principle feasibility of mini-laparoscopy for diagnosing abdominal TB, this study was not designed to identify patient subgroups who benefit from mini-laparoscopy or to compare mini-laparoscopy to other techniques like conventional laparoscopy or image-guided puncture techniques. For example, mini-laparoscopy may have limitations in cases in which suspected lesions are not easily accessible. Secondly, since no standardized study protocol was used, not all microbiological, histopathological, and radiological analyses were performed on all tissue samples in our cohort, so we were not able to assess the diagnostic sensitivity of the different diagnostic methods. Thirdly, the feasibility and relative safety of mini-laparoscopy, which we demonstrated at our tertiary care center with experience with this technique and specialization in infectious diseases may not apply to other healthcare settings. Fourthly, the patient number of this case series is rather small given the low overall incidence of abdominal TB in Germany. Prospective studies on larger patient cohorts including low-resource settings with a higher incidence of TB are needed to establish practical diagnostic algorithms including mini-laparoscopy for patients with suspected abdominal TB.

In conclusion, our study emphasizes that mini-laparoscopy is a safe and valuable tool in patients with suspected abdominal TB that allows visual assessment of the abdominal viscera and peritoneum and guided biopsies from suspicious focal lesions. It has a higher diagnostic yield than radiologic imaging and allows for drug susceptibility testing to select therapy and improve treatment outcomes in patients with drug-resistant tuberculosis. Regular and timely use of this diagnostic modality could prevent unnecessary procedures or invasive surgery and improve patient outcomes.

## Supplementary Information

Below is the link to the electronic supplementary material.

Supplementary file1 (DOCX 15 kb)

Supplementary file2 (DOCX 23 kb)

Supplementary file3 (DOCX 14 kb)
