# Supplementary material for: Mini-laparoscopy as a diagnostic tool for abdominal tuberculosis: a retrospective series of 29 cases
Source: Surg Endosc. 2022 Oct 13;37(3):1830–7. doi: 10.1007/s00464-022-09703-y (PMC9560738; doi:10.1007/s00464-022-09703-y)
Supplement: Supplementary file 1 — Supplementary file1 (DOCX 15 kb) [file 464_2022_9703_MOESM1_ESM.docx]

**Supplemental table 1 – Laboratory results**

|  | **Normal range** | **Median (IQR)** | **Below normal range, n (%)** | **Above normal range, n (%)** |
| --- | --- | --- | --- | --- |
| Hemoglobin (g/dL) | 12.3-15.3 | 10.9 (9.7-11.6) | 24 (83) |  |
| Leukocytes (x10^9^/L) | 3.8-11.0 | 5.9 (4.5-7.6) | 4 (14) | 2 (7) |
| Platelets (x10^9^/L) | 150-400 | 328 (219-390) | 1 (3) | 7 (24) |
| CrP (mg/L) | ≤ 5 | 92 (67-115) |  | 29 (100) |
| ESR (mm/h) | ≤ 20 | 59 (36-77) |  | 20 (69) |
| ASAT (U/L) | 10-35 | 35 (28-82) |  | 14 (48) |
| ALAT (U/L) | 10-35 | 31 (18-56) |  | 12 (41) |
| GGT (U/L) | ≤ 38 | 80 (46-168) |  | 24 (83) |
| AP (U/L) | 35-104 | 105 (86-169) |  | 13 (45) |
| Albumin (g/L) | 35-50 | 29 (24-32) | 18 (62) |  |
| Serum creatinine (mg/dL) | 0.5-1.2 | 0.9 (0.8-1.0) |  | 4 (14) |
| Urea (mg/dL) | 7-19 | 10 (9-17) |  | 6 (21) |
| LDH (U/L) | 84-246 | 323 (263-393) |  | 20 (69) |
| INR | 0.9-1.1 | 1.1 (1.0-1.2) |  | 14 (48) |

### IQR, interquartile range; CrP, C-reactive protein; ESR, erythrocyte sedimentation range; ASAT, aspartate aminotransferase; ALAT, alanine aminotransferase; GGT, gamma-glutamyl transpeptidase; AP, alkaline phosphatase; LDH, lactate dehydrogenase; INR, international normalized ratio
