# Supplementary material for: Mini-laparoscopy as a diagnostic tool for abdominal tuberculosis: a retrospective series of 29 cases
Source: Surg Endosc. 2022 Oct 13;37(3):1830–7. doi: 10.1007/s00464-022-09703-y (PMC9560738; doi:10.1007/s00464-022-09703-y)
Supplement: Supplementary file 2 — Supplementary file2 (DOCX 23 kb) [file 464_2022_9703_MOESM2_ESM.docx]

|  | **Diag** | **Liver** | | | | | | | **Peritoneum** | | | | | | | **Spleen** | | | | | | |
| --- | --- | --- | --- | --- | --- | --- | --- | --- | --- | --- | --- | --- | --- | --- | --- | --- | --- | --- | --- | --- | --- | --- |
|  |  | **ML** | **US** | **CT** | **AFB stain** | **Cul-ture** | **PCR** | **Histo-logy** | **ML** | **US** | **CT** | **AFB stain** | **Cul-ture** | **PCR** | **Histo-logy** | **ML** | **US** | **CT** | **AFB stain** | **Cul-ture** | **PCR** | **Histo-logy** |
| **1** | H | - | - | - | - | - | - | nCG | + | / | - | / | / | / | CG | - | + | + | - | - | - | CG |
| **2** | C | + | - | - | - | - | - | nCG | - | / | - | / | / | / | / | 0 | - | - | / | / | / | / |
| **3** | M | + | - | - | - | + | - | nCG | + | / | - | - | + | + | CG | + | - | - | / | / | / | / |
| **4** | M | + | + | - | - | - | / | - | + | / | - | - | + | + | CG | - | + | - | / | / | / | / |
| **5** | C | + | - | - | - | - | - | nCG | + | / | - | - | - | - | nCG | 0 | - | - | / | / | / | / |
| **6** | M | + | - | + | - | + | + | CG | - | / | - | / | / | / | / | 0 | + | + | / | / | / | / |
| **7** | M | + | - | - | / | / | / | / | + | / | - | - | + | + | CG | - | - | - | / | / | / | / |
| **8** | M | 0 | - | - | / | / | / | / | + | / | + | - | + | + | CG | 0 | - | - | / | / | / | / |
| **9** | C | + | - | + | - | - | - | I | - | / | - | / | / | / | / | 0 | - | - | / | / | / | / |
| **10** | M | - | - | - | - | + | - | CG | - | / | - | / | / | / | / | + | + | - | / | / | / | CG |
| **11** | M | 0 | - | - | / | / | / | / | + | / | + | / | / | - | CG | 0 | - | - | / | / | / | / |
| **12** | M | 0 | - | - | / | / | / | / | + | / | + | - | + | - | / | 0 | - | - | / | / | / | / |
| **13** | C | + | - | - | - | - | / | - | + | / | + | - | - | - | - | - | - | - | / | / | / | / |
| **14** | M | + | / | - | - | - | - | I | + | / | + | - | + | + | nCG | - | / | - | / | / | / | / |
| **15** | M | 0 | - | / | / | / | / | / | + | / | / | - | + | + | CG | 0 | - | / | / | / | / | / |
| **16** | M | 0 | / | - | / | / | / | / | + | / | - | - | + | + | CG | 0 | / | - | / | / | / | / |
| **17** | C | - | - | - | - | - | - | nCG | - | / | - | / | / | / | / | 0 | - | - | / | / | / | / |
| **18** | C | - | - | - | - | - | / | nCG | - | / | - | / | / | / | / | + | - | - | - | - | / | nCG |
| **19** | C | - | - | + | - | - | - | I | - | / | - | / | / | / | / | + | + | + | - | - | - | I |
| **20** | H | + | / | + | - | - | / | CG | - | / | - | / | / | / | / | 0 | / | + | / | / | / | / |
| **21** | H | + | - | + | - | - | - | CG | - | / | - | / | / | / | / | 0 | + | + | / | / | / | / |
| **22** | M | + | - | - | + | + | + | CG | - | / | - | / | / | / | / | + | + | + | / | / | / | / |
| **23** | M | - | - | / | - | - | + | nCG | - | / | / | / | / | / | / | + | + | / | - | - | - | I |
| **24** | C | 0 | - | / | / | / | / | / | + | / | / | / | / | / | / | 0 | - | / | / | / | / | / |
| **25** | M | - | - | - | / | / | / | I | + | / | - | - | + | + | CG | - | - | - | / | / | / | / |
| **26** | M | + | - | - | - | + | + | nCG | + | / | - | - | + | + | I | - | - | - | / | / | / | / |
| **27** | C | 0 | - | - | / | / | / | / | + | / | - | / | / | / | / | 0 | - | - | / | / | / | / |
| **28** | M | + | - | - | - | + | + | nCG | + | / | - | / | / | / | / | - | - | - | / | / | / | / |
| **29** | C | + | - | + | - | - | - | I | - | / | - | / | / | / | / | - | - | + | / | / | / | / |

**Supplemental Table 2 – Macroscopic, microbiological and histopathological results of all 29 patients diagnosed with abdominal tuberculosis**

H, histopathological diagnosis; C, clinical diagnosis; M, microbiological diagnosis, ML, mini-laparoscopy; US, ultrasound; CT, computed tomography; AFB, acid-fast bacilli; PCR, polymerase chain reaction; nCG, non-caseating granulomas; CG, caseating granulomas; I, inflammation
