# Supplementary material for: Mini-laparoscopy as a diagnostic tool for abdominal tuberculosis: a retrospective series of 29 cases
Source: Surg Endosc. 2022 Oct 13;37(3):1830–7. doi: 10.1007/s00464-022-09703-y (PMC9560738; doi:10.1007/s00464-022-09703-y)
Supplement: Supplementary file 3 — Supplementary file3 (DOCX 14 kb) [file 464_2022_9703_MOESM3_ESM.docx]

**Supplemental table 3 – Post-biopsy bleeding**

|  | **Total** | **No bleeding** | **Spontanous resolution** | **APC** | **APC + fibrin glue** |
| --- | --- | --- | --- | --- | --- |
| **Liver** | 29 | 5 | 13 | 11 |  |
| **Spleen** | 5 |  |  | 4 | 1 |
| **Peritoneum** | 17 | 16 | 1 |  |  |

APC, argon-plasma coagulation
